# Supplementary material for: Navigating the maze of mass spectra: a machine-learning guide to identifying diagnostic ions in O-glycan analysis
Source: Anal Bioanal Chem. 2024 Aug 24;417(5):931–43. doi: 10.1007/s00216-024-05500-9 (PMC11782297; doi:10.1007/s00216-024-05500-9)
Supplement: Supplementary file 1 — Supplementary file1 (DOCX 3282 KB) [file 216_2024_5500_MOESM1_ESM.docx]

**Navigating the Maze of Mass Spectra: A Machine-Learning Guide to Identifying Diagnostic Ions in O-Glycan Analysis**

James Urban^1^, Roman Joeres^1,2,3^, Luc Thomès^4^, Kristina A. Thomsson^5^, Daniel Bojar^1,*^

^1^Department of Chemistry and Molecular Biology, University of Gothenburg, Gothenburg, Sweden. Wallenberg Centre for Molecular and Translational Medicine, University of Gothenburg, Gothenburg, Sweden.

^2^Helmholtz Institute for Pharmaceutical Research Saarland, Helmholtz Center for Infection Research, Saarbruecken, Germany

^3^Center for Bioinformatics, Saarland University, Saarbruecken, Germany

^4^University Lille, CHU Lille, ULR 7364 - RADEME - Maladies RAres du DÉveloppement embryonnaire et du Métabolisme, 59000 Lille, France.

^5^Proteomics Core Facility at Sahlgrenska Academy, University of Gothenburg, Gothenburg, Sweden

^*^Corresponding author: daniel.bojar@gu.se

**Supplementary Figures**

**
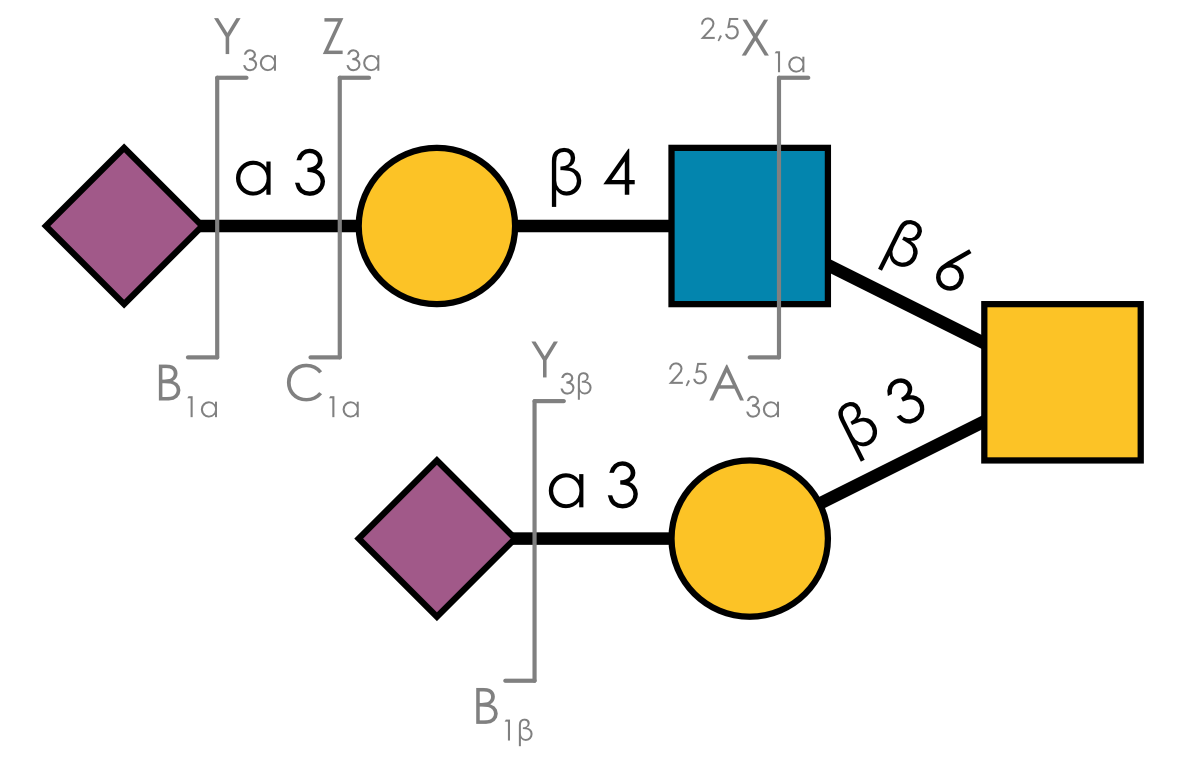
Supplementary Figure 1. Fragmentation pattern of glycans in mass spectrometry.** Breaking points are indicated by gray lines, labeled by the corresponding fragment in Domon-Costello nomenclature. Fragments indicating the left part of the molecule are labeled at the bottom of the break, while fragments indicating the right part of the molecule are labeled at the top. The inclusion of the glycosidic linkage oxygen in the fragment is indicated by the position of the linkage. The glycan is depicted via the Symbol Nomenclature for Glycans.


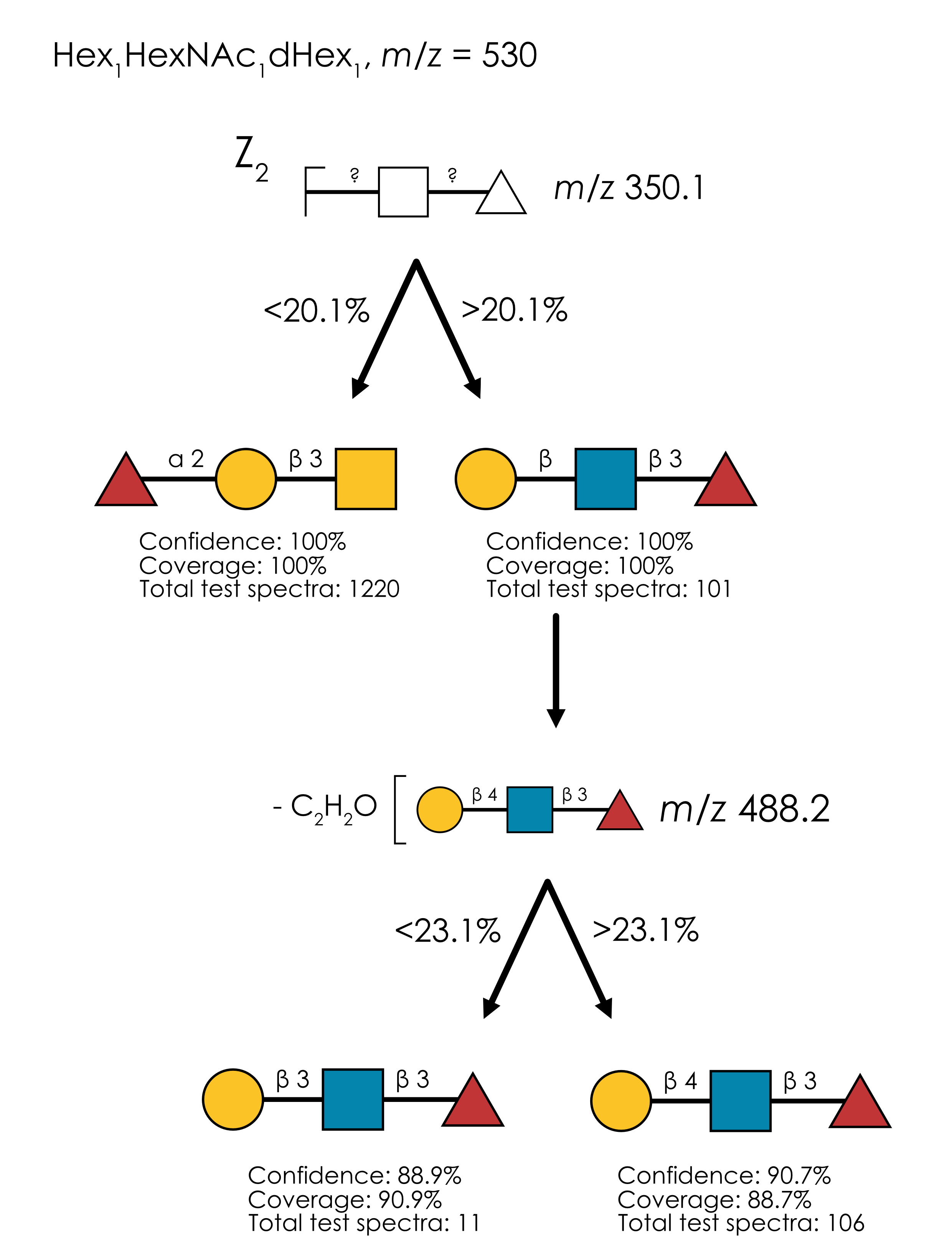


**Supplementary Figure 2. Learned annotation rules for Hex_1_HexNAc_1_dHex_1_ (*m/z* 530).** Using our rule-based machine learning approach, we present the best splitting rules for distinguishing isomers of this composition. Thresholds are provided as % of the maximum intensity peak or as ratio values.

**
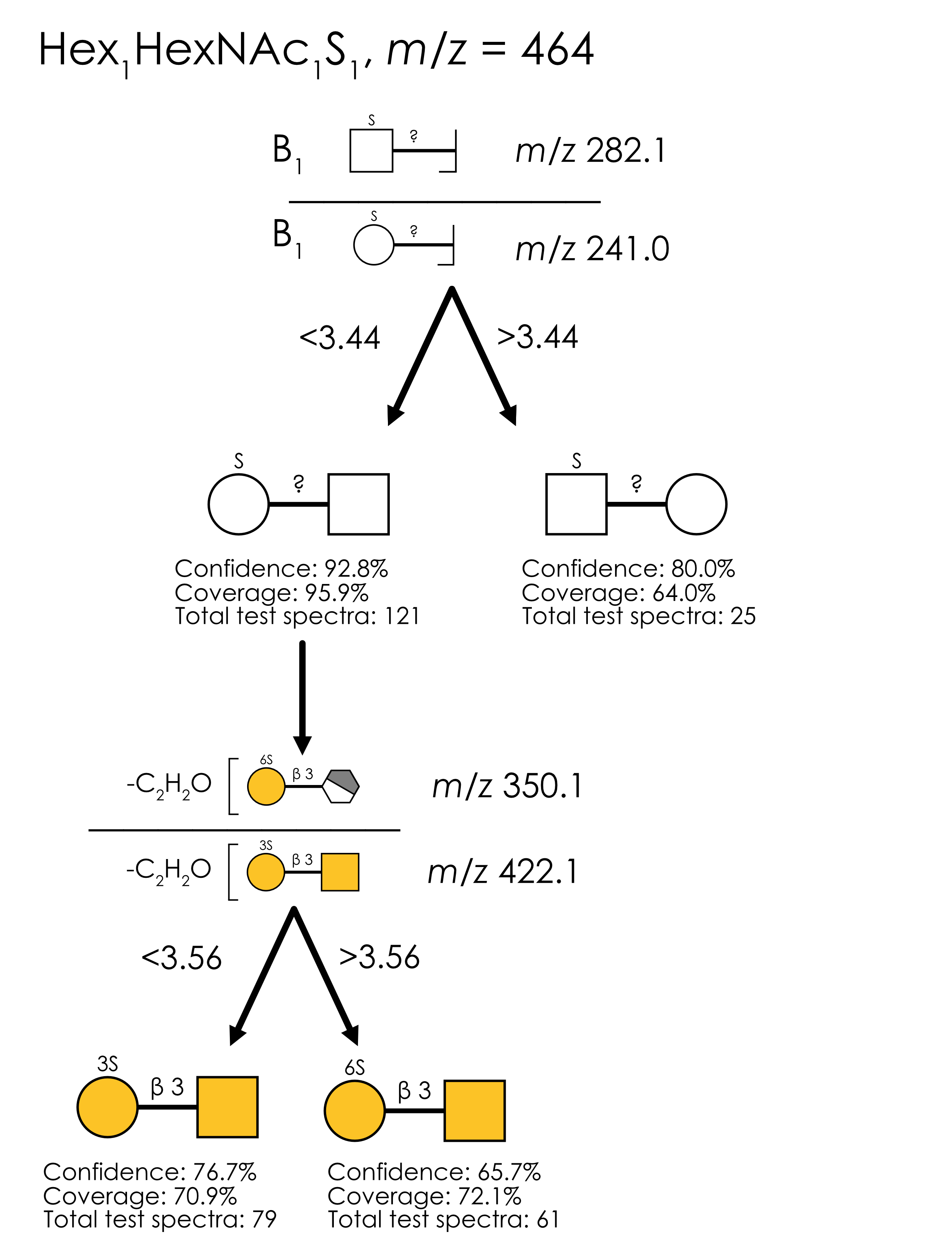
**

**Supplementary Figure 3. Learned annotation rules for Hex_1_HexNAc_1_S_1_ (*m/z* 464).** Using our rule-based machine learning approach, we present the best splitting rules for distinguishing isomers of this composition. Thresholds are provided as % of the maximum intensity peak or as ratio values.

**
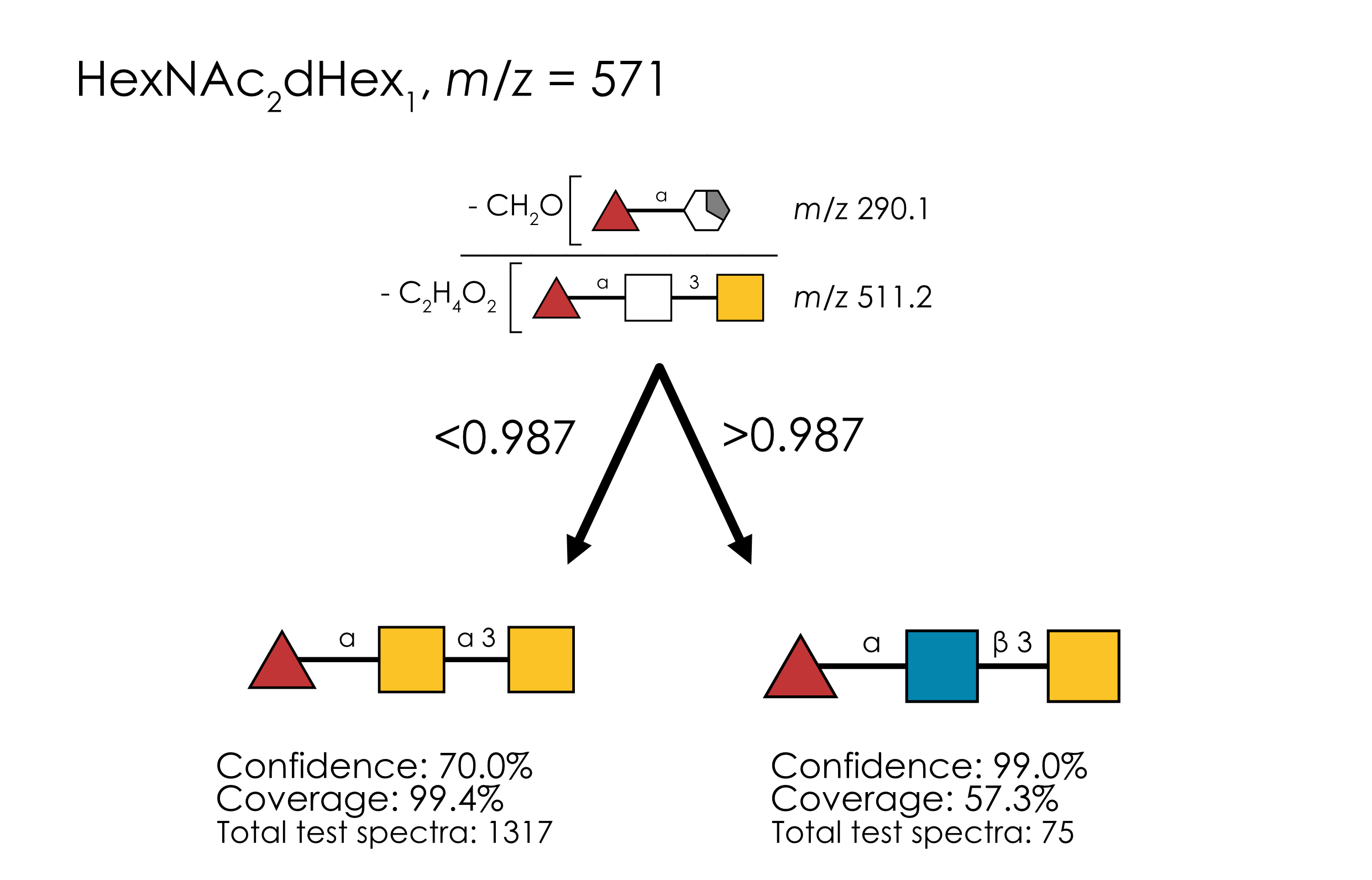
**

**Supplementary Figure 4. Learned annotation rules for HexNAc_2_dHex_1_ (*m/z* 571).** Using our rule-based machine learning approach, we present the best splitting rules for distinguishing isomers of this composition. Thresholds are provided as % of the maximum intensity peak or as ratio values.

**
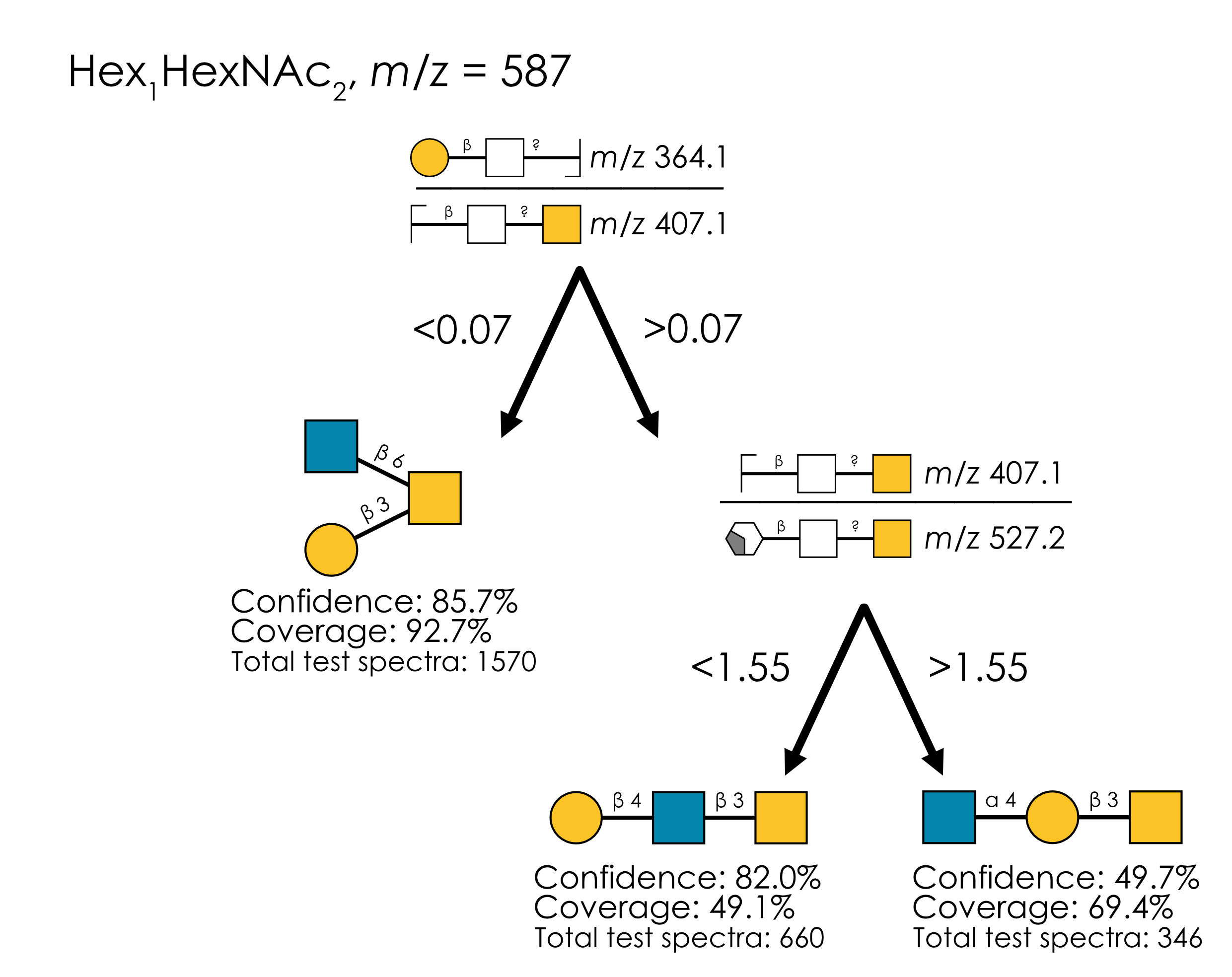
**

**Supplementary Figure 5. Learned annotation rules for Hex_1_HexNAc_2_ (*m/z* 587).** Using our rule-based machine learning approach, we present the best splitting rules for distinguishing isomers of this composition. Thresholds are provided as % of the maximum intensity peak or as ratio values.


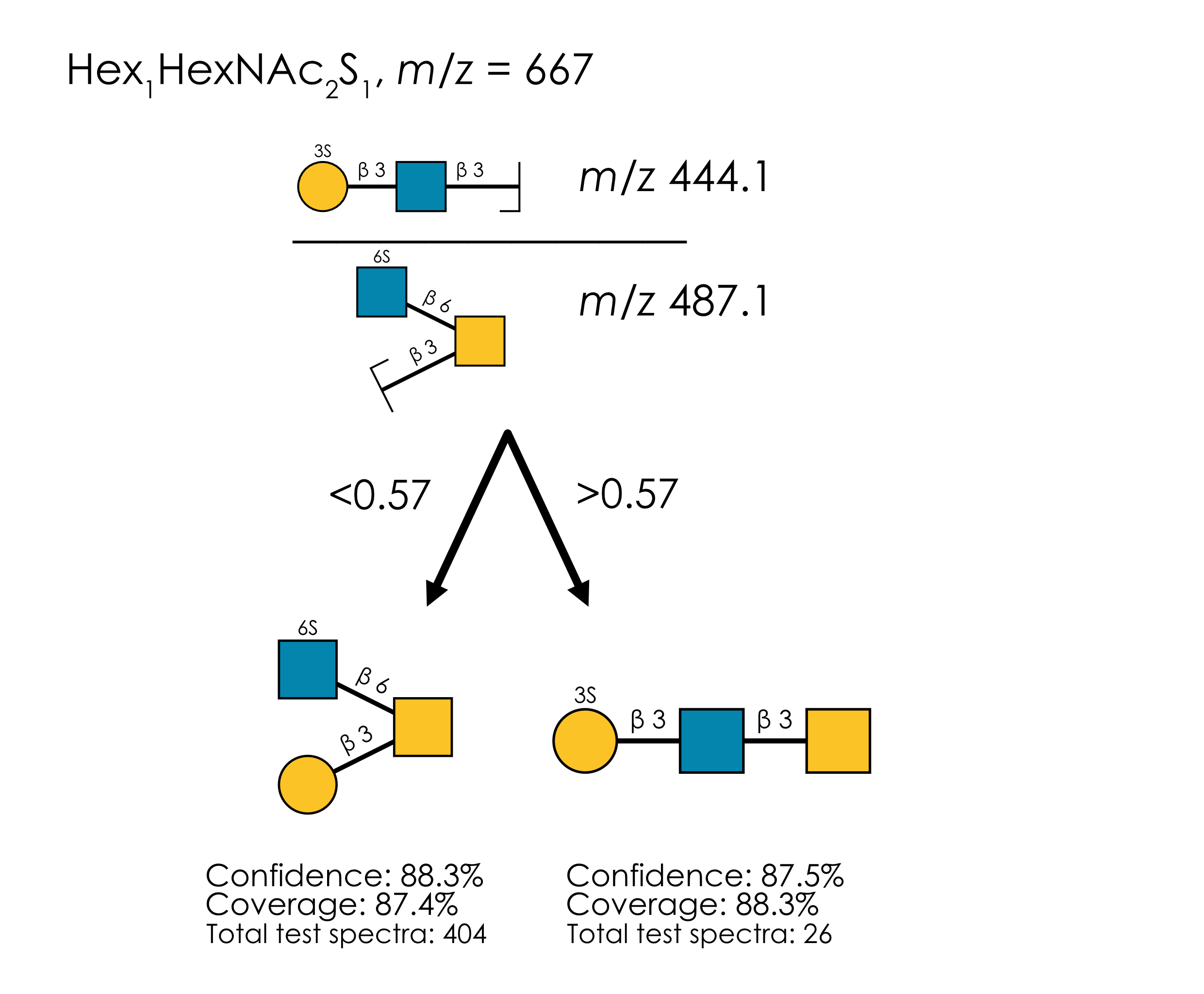


**Supplementary Figure 6. Learned annotation rules for Hex_1_HexNAc_2_S_1_ (*m/z* 667).** Using our rule-based machine learning approach, we present the best splitting rules for distinguishing isomers of this composition. Thresholds are provided as % of the maximum intensity peak or as ratio values.


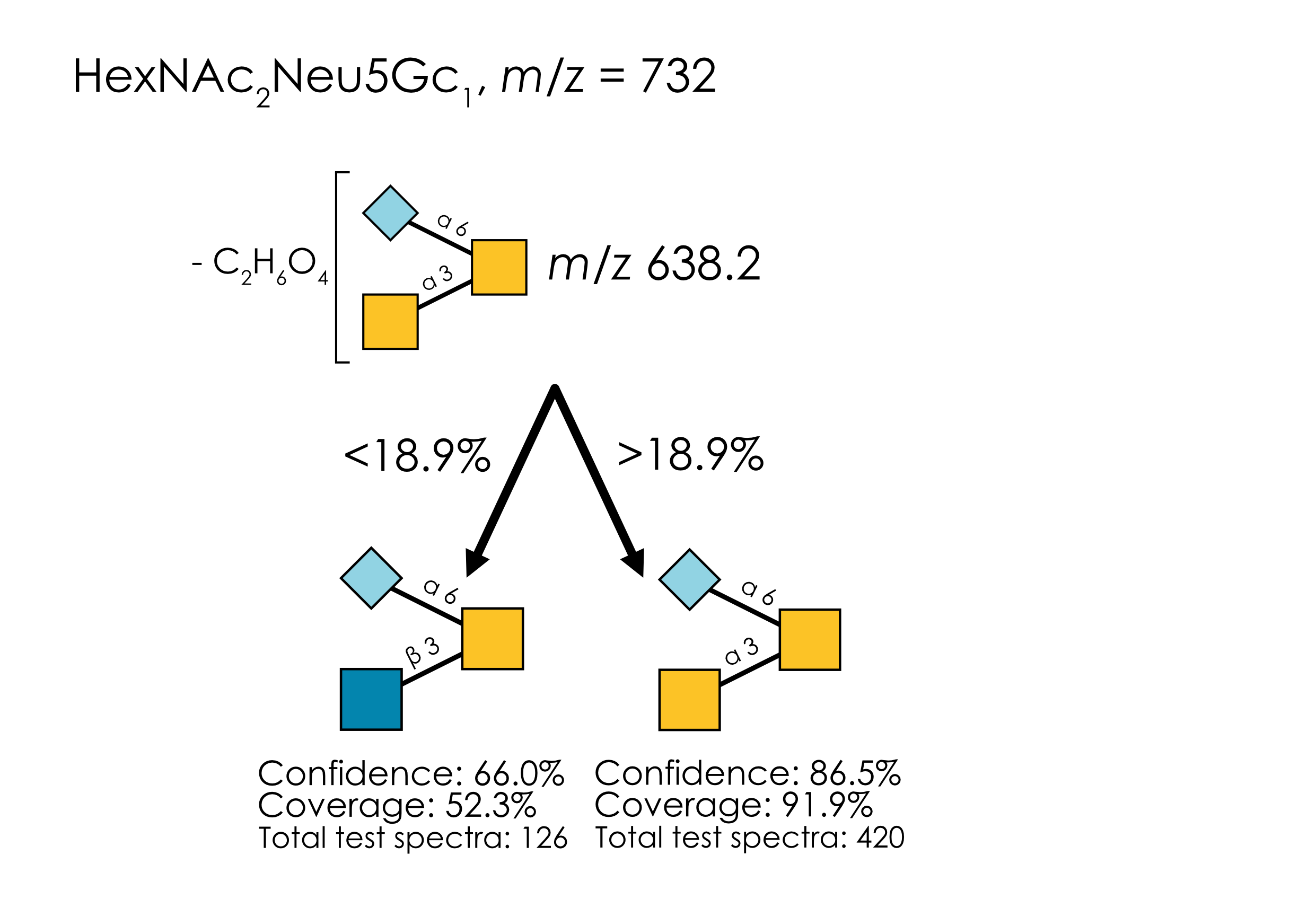


**Supplementary Figure 7. Learned annotation rules for HexNAc_2_Neu5Gc_1_ (*m/z* 732).** Using our rule-based machine learning approach, we present the best splitting rules for distinguishing isomers of this composition. Thresholds are provided as % of the maximum intensity peak or as ratio values.


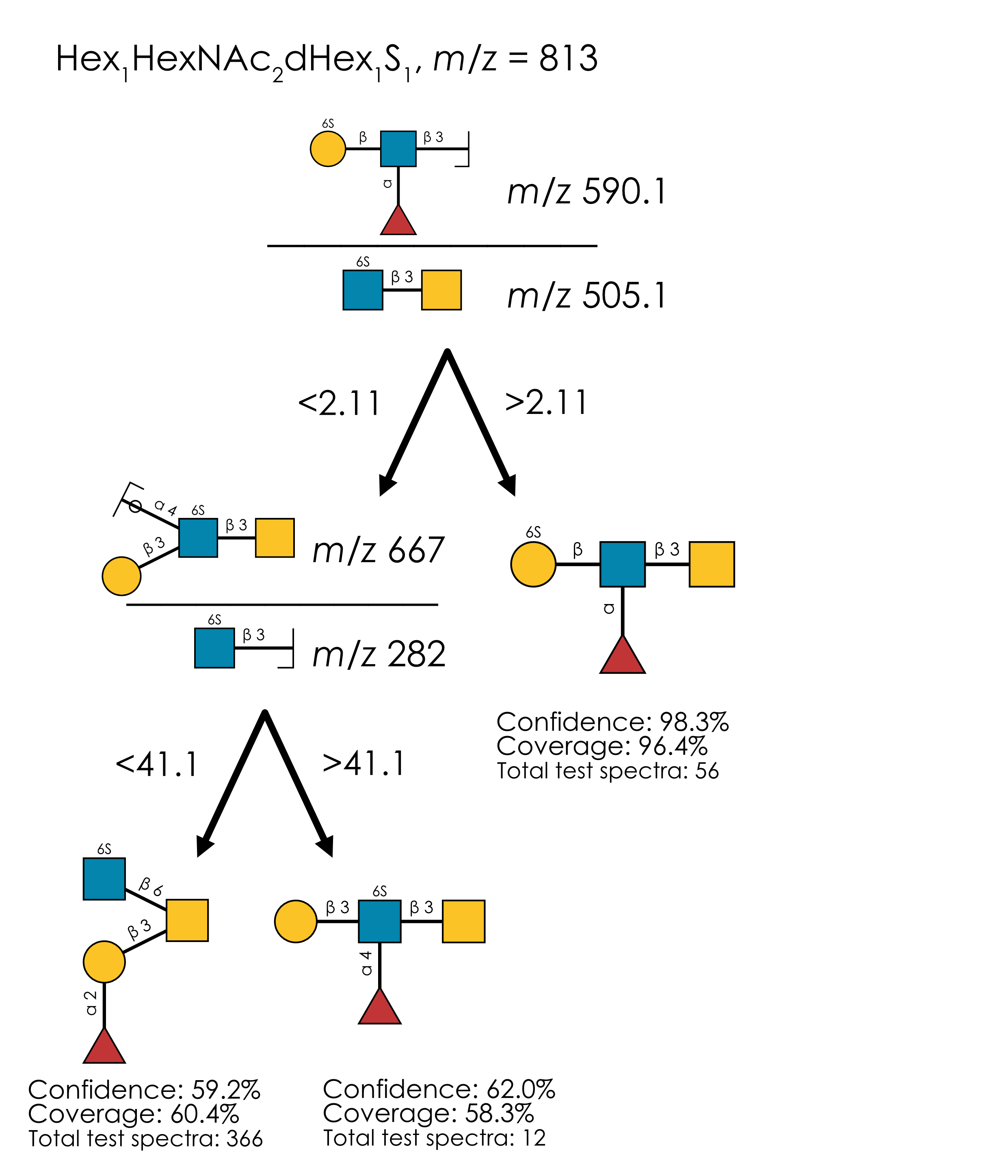


**Supplementary Figure 8. Learned annotation rules for Hex_1_HexNAc_2_dHex_1_S_1_ (*m/z* 813).** Using our rule-based machine learning approach, we present the best splitting rules for distinguishing isomers of this composition. Thresholds are provided as % of the maximum intensity peak or as ratio values.


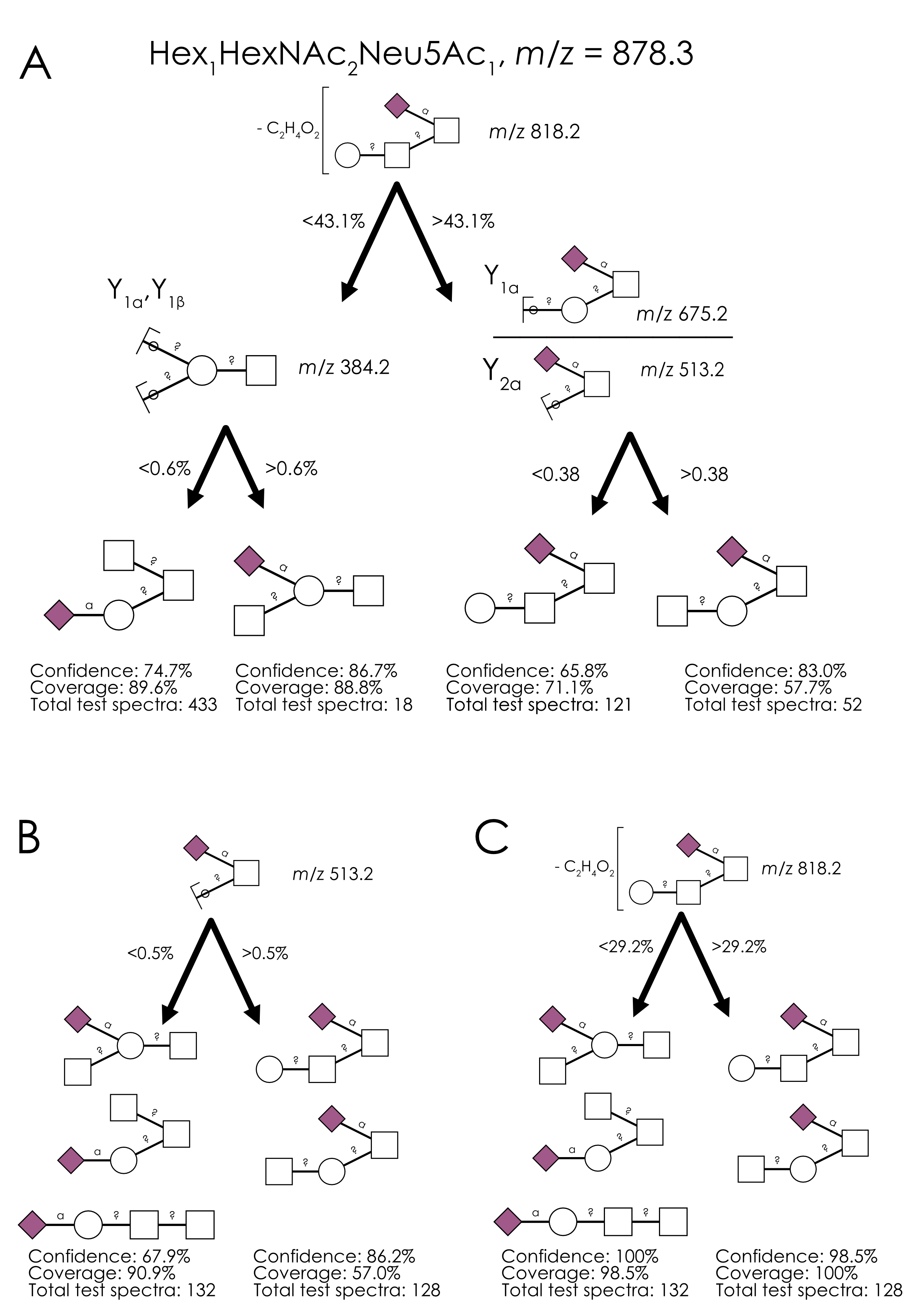


**Supplementary Figure 9. Annotating Hex_1_HexNAc_2_Neu5Ac_1_ (*m/z* 878.3) with different diagnostic fragments. a-c)** Using our rule-based machine learning approach, we present the best splitting rules for distinguishing isomers of this composition. Thresholds are provided as % of the maximum intensity peak or as ratio values. The best model used M-C_2_H_2_O-H_2_O (*m/z* 818.2) as the topological distinguisher (a), which achieved higher confidence than using the traditional *m/z* 513.2 fragment (b). We further showed that using the M-C_2_H_2_O-H_2_O fragment (*m/z* 818.2), indicative of Sia-HexNAc in many other isomers, also achieved a higher average confidence and coverage than the aforementioned *m/z* 513.2 fragment (c).


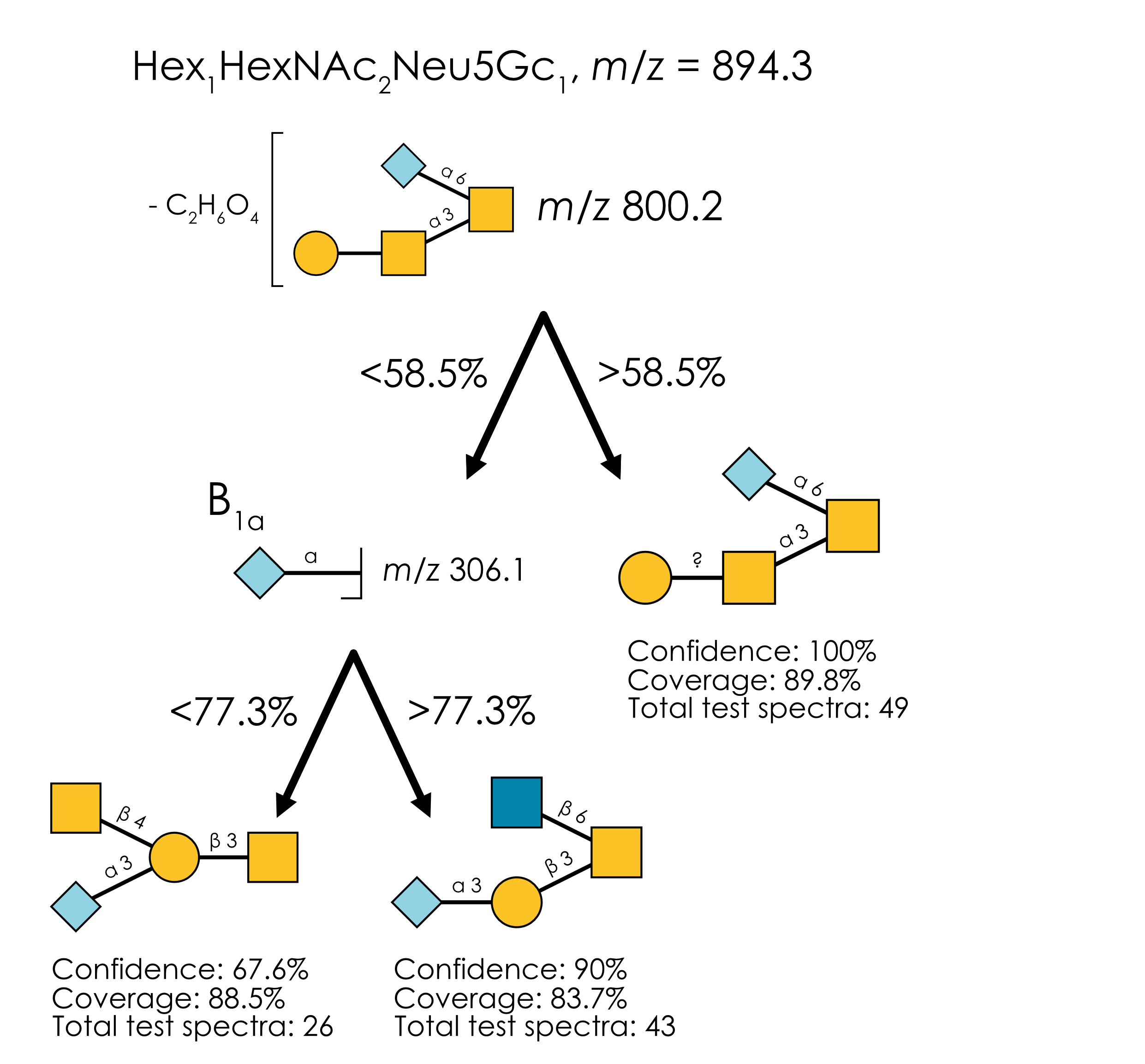


**Supplementary Figure 10. Learned annotation rules for Hex_1_HexNAc_2_Neu5Gc_1_ (*m/z* 894.3).** Using our rule-based machine learning approach, we present the best splitting rules for distinguishing isomers of this composition. Thresholds are provided as % of the maximum intensity peak or as ratio values.


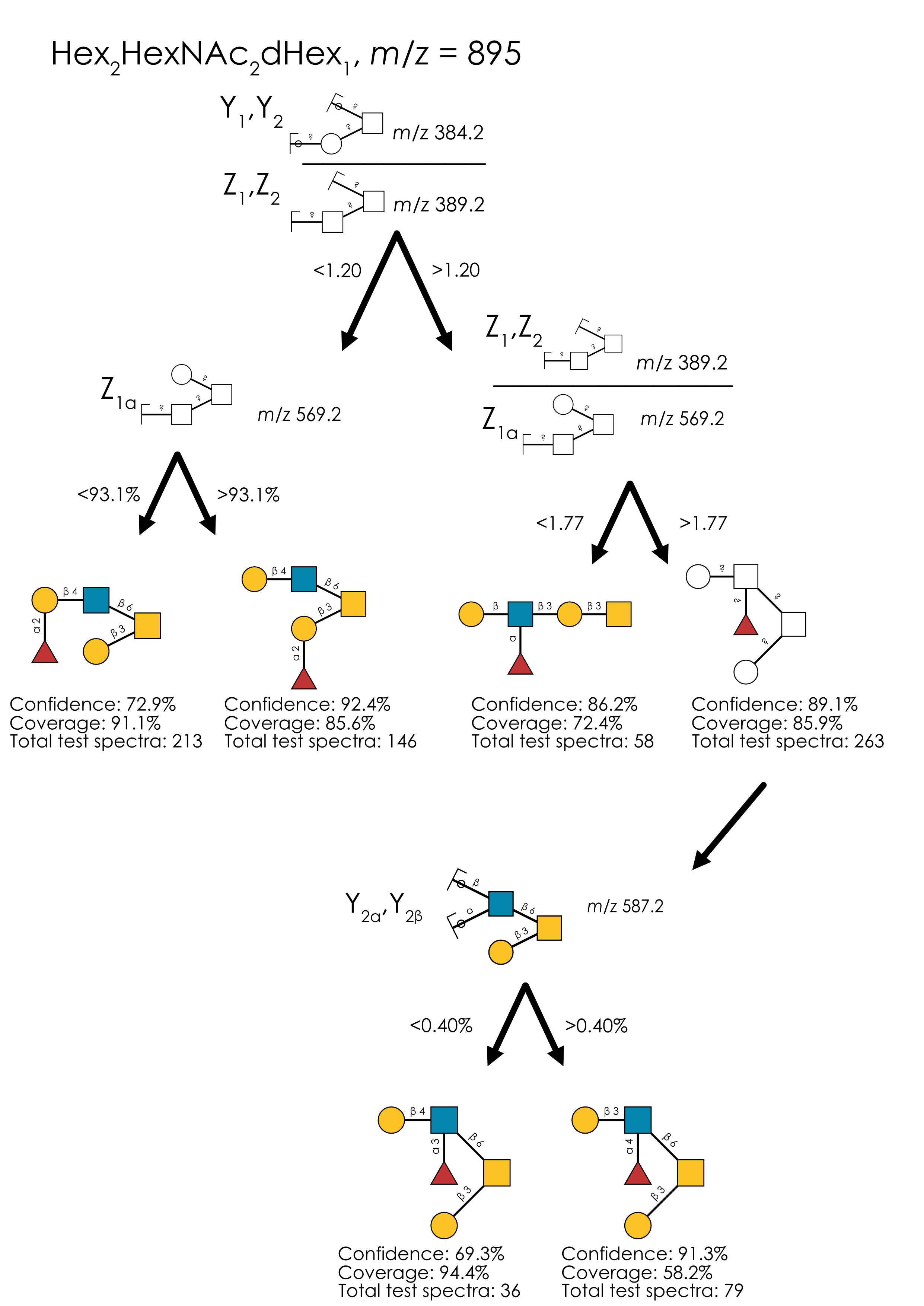


**Supplementary Figure 11. Learned annotation rules for Hex_2_HexNAc_2_dHex_1_ (*m/z* 895).** Using our rule-based machine learning approach, we present the best splitting rules for distinguishing isomers of this composition. Thresholds are provided as % of the maximum intensity peak or as ratio values.

**
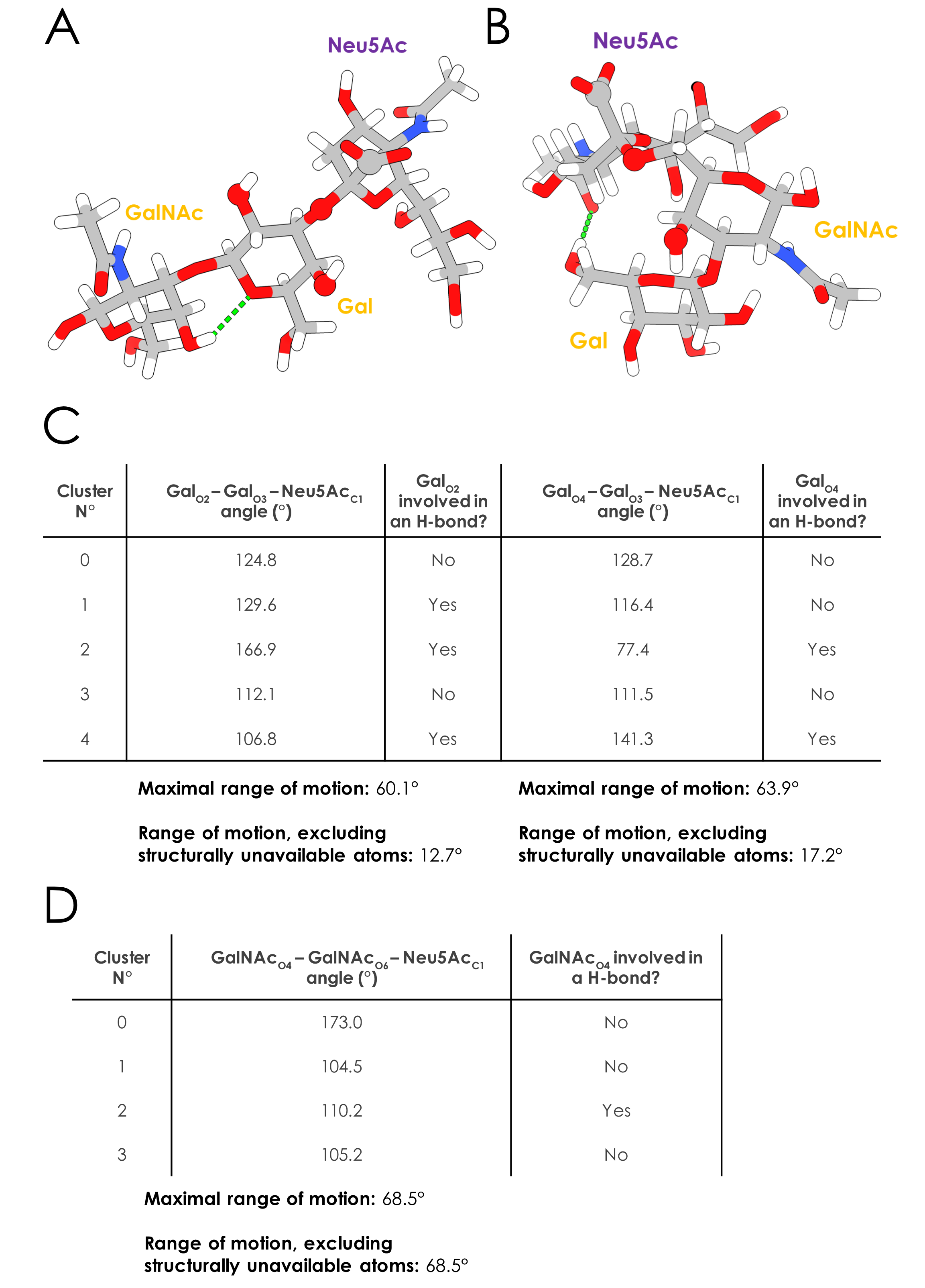
**

**Supplementary Figure 12. Structural flexibility of Hex_1_HexNAc_1_Neu5Ac_1_ isomers. A-B)** Representative 3D structures of Neu5Acα2-3Galβ1-3GalNAc (A) and Galβ1-3(Neu5Acα2-6)GalNAc (B) from GlycoShape. Green dotted lines represent predicted hydrogen bonds. Gal_O2_, Gal_O3_, and Gal_O4_ (in A), GalNAc_O4_ and GalNAc_O6_ (in B) together with Neu5Ac_C1_, used to compute torsion angles, are depicted with larger spheres. **C-D)** Torsion angles from all structural clusters of Neu5Acα2-3Galβ1-3GalNAc (C) and Galβ1-3(Neu5Acα2-6)GalNAc (D). Participation of Gal_O2_ and Gal_O4_ (C) and GalNAc_O4_ (D) in predicted hydrogen bonds is also presented, impacting structural availability.


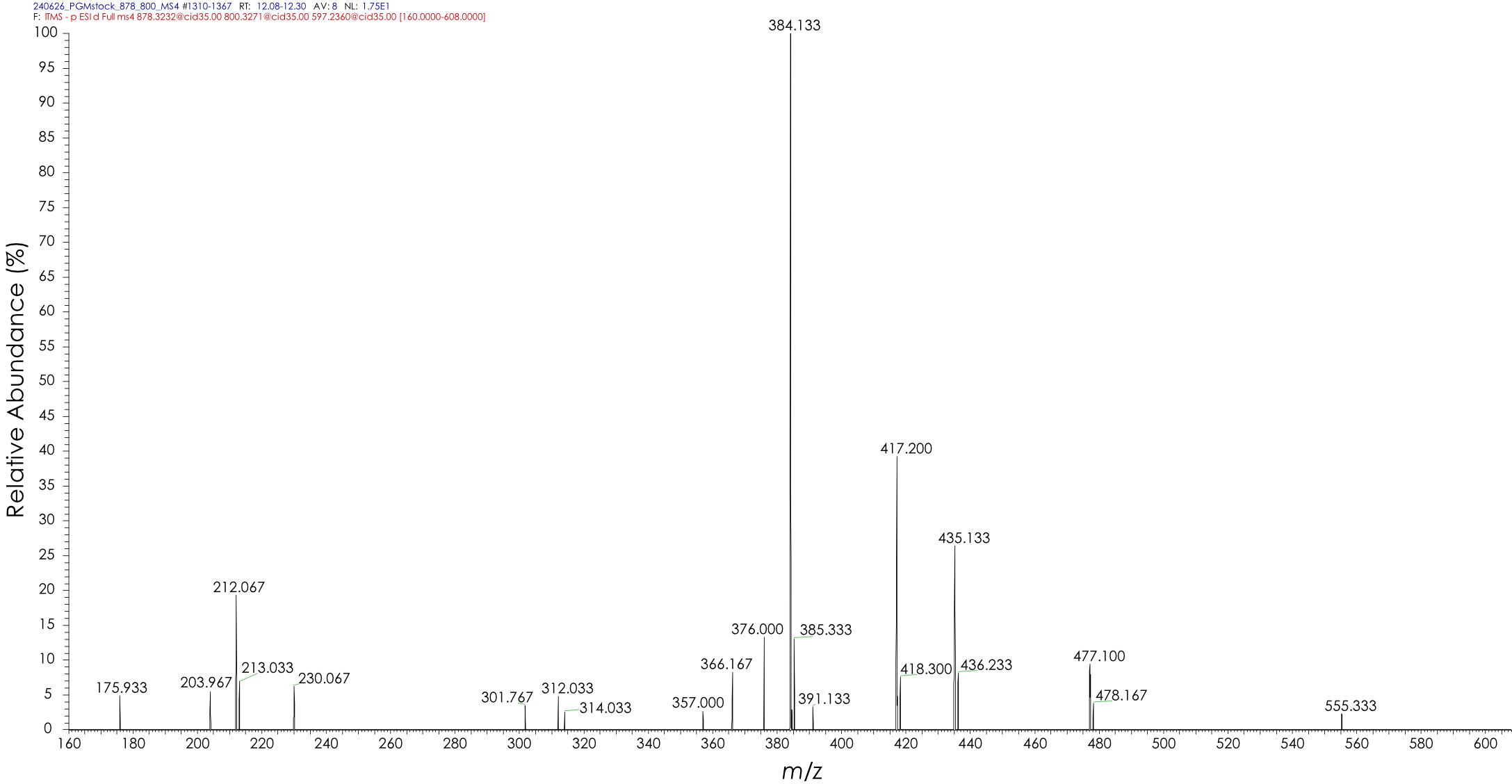


**Supplementary Figure 13. Similarity of HexNAc?1-?Galβ1-3(Neu5Acα2-6)GalNAc and Galβ1-3(Neu5Acα2-6)GalNAc diagnostic fragments.** MS^4^ spectrum of *m/z* 597 (-C_2_H_6_O_3_ -HexNAc) fragment taken from the fragmentation of the *m/z* 800 (-C_2_H_6_O_3_) fragment produced by HexNAc?1-?Galβ1-3(Neu5Acα2-6)GalNAc in porcine gastric mucin.
